# Smart control of soil temperature to optimize root-zone conditions for enhancing the physiological performance, growth, and productivity of greenhouse-grown cucumber

**DOI:** 10.1038/s41598-026-40825-8

**Published:** 2026-03-26

**Authors:** K. M. Refaie, Shereen A. H. Saad, Nermin S. Hussein

**Affiliations:** 1 Central Laboratory for Agricultural Climate, Agricultural Research Center, Dokki, Giza, Egypt; 2 Physics and Chemistry Department, Soils, Water and Environment Research Institute, Agricultural Research Center, Giza, Egypt; 3 Agricultural Engineering Research Institute, Agricultural Research Center, Dokki, Giza, Egypt

**Keywords:** *Cucumis sativus*, Smart management techniques, Soil temperature, Plant photosynthesis, Vapor pressure deficit, Cucumber growth, Yield, Ecology, Ecology, Environmental sciences, Plant sciences

## Abstract

Determining the optimal soil temperature in the active root zone to meet the requirements of different plant growth stages is crucial. Controlled greenhouse producers attempt to heat soil sectors in various ways without determining an appropriate or reliable soil temperature. A suitable smart method can be used to adjust the temperature in the active root zone to determine the optimal temperature and improve crop growth and yield. Cucumber plants grown in a plastic greenhouse were subjected to five different soil temperature treatments (13 as a control, 16, 19, 22, and 25 °C) over two growing seasons using carbon fiber smart thermal cable connected to a metal thermostat. The thermal cable was installed 10 cm below the soil surface, extended 20 m apart on three branches per replicate, and then buried and compacted well into the soil. The objective of this study was to determine the optimum soil temperature by smart thermal system, to enhance photosynthesis rate, growth parameters, and yield of cucumber, as well as improving the availability of nutrients within active root zone, and to minimize vapor pressure deficit. The experimental design was a completely randomized design with five treatments and three replicates. The smart soil heating system was a feasible economic option, with a benefit–cost ratio of 1.75% versus 1.1% for the traditional system, resulting in improved cucumber yield and quality, while saving approximately 30% of energy. Plant photosynthesis was positively correlated with total yield but negatively correlated with vapor pressure deficit, suggesting that the initial or mid-season increases in cucumber yield were partly due to the increased soil temperature at 22 °C. In conclusion, plants grown at 19 or 22 °C had significantly similar yields of 3.37 ± 0.09 and 3.71 ± 0.14 kg plant^−1^, respectively, and most of these plants had higher yields than plants grown at 16 °C, with a yield of 2.33 ± 0.03 kg plant^−1^. We suggest that a soil temperature of 22 °C is ideal for cucumber growers in the initial or mid-season, but at the end of the season the plant may not be affected, so a soil temperature of 19 °C can be used to save energy. Also, the possibility of presenting this intelligent system as an alternative the traditional one.

## Introduction

The unfavorable soil temperature, whether natural or artificial, severely affects crops’ growth and productivity. Due to the unavailability of nutrients and the lack of movement within different soil profiles, or the weak of plant growth and dwarfing of the root system that prevents it from penetrating and distributed into the soil^1,2^. Furthermore, this may have physiological effects, such as a decrease or cessation of metabolic processes important for growth and productivity. Furthermore, this can induce physiological stress, leading to reduced or even inhibited metabolic processes essential for plant growth and productivity. Moreover, low soil temperatures stimulate soil-borne diseases and reduce the water and nutrient uptake ability of roots, particularly the uptake of phosphorus^2–4^. Soil temperature is crucial, also in particular at germination and the young plant stage. If the soil temperature remains low for a long time, the plants are negatively affected. For this reason, cucumber is said to need a “warm foot”. Soil heating enables cucumber plants to better endure low air temperature^3^.

Innovative agricultural technologies, particularly in cucumber (*Cucumis sativus* L.) production, represent a promising means of improving productivity, quality, and sustainability. There is a growing need for smart integrated management techniques, both in protected and open-field cultivation, as well as advanced methods to meet food demand^5^. This is due to the availability of highly sensitive sensor technologies for monitoring climate factors, plant nutrients, soil moisture, precision irrigation, heating (air and soil), as well as shading and ventilation. The results of automated control practices in smart irrigation systems are a clear example of reducing water and fertilizer requirements and contributing to improved overall resource use. Furthermore, their effectiveness is reflected in the development and empowerment of these systems^6^. Emphasis should be placed on renewable and intelligent energy-efficient control systems in Mediterranean greenhouses, where crops such as tomatoes and cucumbers are widely grown^7^.

Cucumber is one of the major vegetable crops cultivated under protected cultivation in Egypt^8^ It is a sub-tropical vegetable crop that grows successfully under conditions of high light, high humidity, high soil moisture, temperature and fertilizers in greenhouses^9,10^. Therefore, the importance of studying the soil temperature is very important to maintain the growth and productivity of the cucumber crop in addition to reducing the waste of energy and working to achieve a green economy and sustainable production, especially with the expansion of the construction of the mega project of protected cultivations in line with Egypt’s Sustainable Plan 2030.

The main objective of this study was to intelligently determine the optimal soil temperature to enhance photosynthesis and improve nutrient availability in the soil, improve cucumber growth and productivity, and help greenhouse producers save energy. Furthermore, this intelligent system was presented as an alternative to traditional methods under similar climatic conditions.

## Materials and methods

**Fig. 1:**
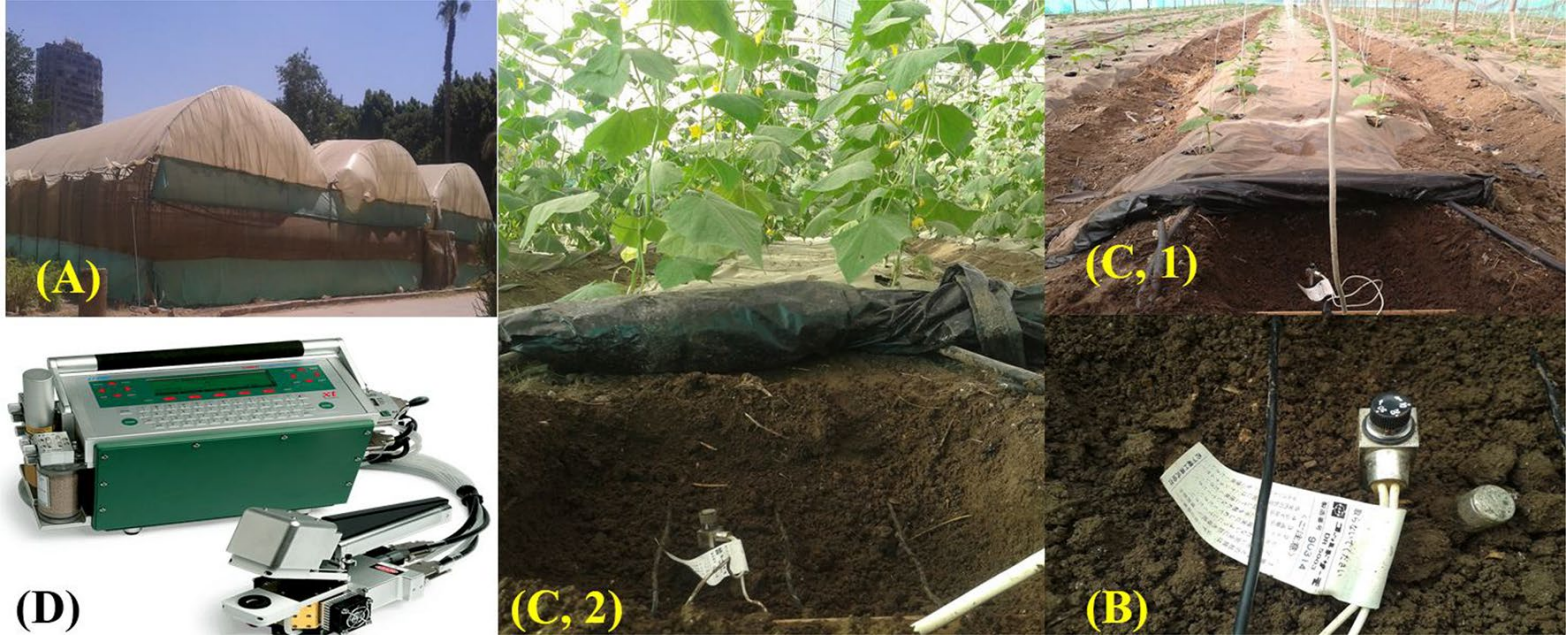
Modified treble-span greenhouse (**A**), metallic thermostat with protective cover (**B**), thermal cable installed in the soil at different plant growth stages (**C1** and **C2**), and a portable photosynthesis system (LI-6400, LI-COR Biosciences, USA) (**D**).

The present investigation was carried out in Dokki site El-Giza governorate, Egypt which situated at 30° 03ʹ N latitude, 31° 20ʹ E longitude. This study was established during the two winter seasons of 2021 and 2022. The seedlings were transplanted on October 24, 2021, and October 28, 2022, respectively, after cucumber seeds (*Cucumis sativus* L. cv. DP 161 F_1_) had been sown in the nursery three weeks earlier. A modified of triple-span style of greenhouse (Fig. 1-A) was constructed covering 1620 m^2^, with 4.5 m height, 25.5 m width and 60 m long. Each single one of greenhouse was divided into 5 beds with 1 m wide are covered by a black plastic mulch of 80 microns thickness.

The cultivation at the Egyptian Protected Cultivation Center in Dokki, Agricultural Research Center (ARC), Giza Governorate, Egypt. Table 1 summarizes the monthly climatological normal data for the open field and greenhouses at the experimental site. Based on the temperature data, soil temperature indices were determined. The data also shows the evapotranspiration rate (Et_o_) in the open field, calculated using the Penman–Monteith equation and modified for greenhouses, according to^11^ which were used to determine the amount of irrigation water. Drip irrigation was an irrigation system, with emitters built in laterals tubes of two liter per hour at one bar operating pressure. A mixture of (100 kg of calcium super phosphate, 25 kg of ammonium nitrate, 50 kg of potassium sulfate, 15 kg of sulfur, and 5 m^3^ of compost per 540 m^3^ in one single greenhouse area) was added as a starter, two weeks before transplanting. All the other agriculture practices were performed according to the standard recommendations for commercial growers by^12^. The physical and chemical analyses of the soil, before adding the mixed fertilizer were presented in Table 2 that estimated according to^13^.

**Table 1:** Monthly climatological normal data in the open field and under greenhouse conditions at the Giza–Dokki experimental site.

Parameters		Open fild					Greenhouse				
		Oct	Nov	Dec	Jan	Feb	Oct	Nov	Dec	Jan	Feb
Temp. C°	Max	30.5	25.8	21.3	19.9	21.5	31.0	27.7	21.9	22.1	23.3
	Min	16.2	12.1	8.0	6.2	6.9	20.4	17.0	11.2	11.0	10.3
RH_AVG %		61	69	68	66	59	58	68	66	68	66
Leaf wetness (min)		446	613	276	364	412	599	681	278	383	417
Soil temperature °C (min)		12.7	16.0	9.0	10.7	11.0	17.6	11.7	12.5	11.8	12.8
Et_o_ mm day^−1^		1.60	1.90	1.28	1.48	1.85	1.12	1.33	0.91	0.84	1.26

Source: Ground weather stations, Ministry of Civil Aviation-Meteorological Authority, Cairo for Open fild data and The Central Laboratory for Agricultural Climate, Agricultural Research Center, Giza for Greenhouse data.

**Table 2:** Physical and chemical properties of the soil of the experimental area at the Giza–Dokki experimental site.

Texture	Sand (%)	Silt (%)	Clay (%)	Bulk density (g m^−3^)	EC 1:5 (dS m^−1^)	pH (1:2.5)	Total CaCO_3_ (%)
Clay	38.6	4.3	57.1	1.2	3.2	7.9	3.6

**Fig. 2:**
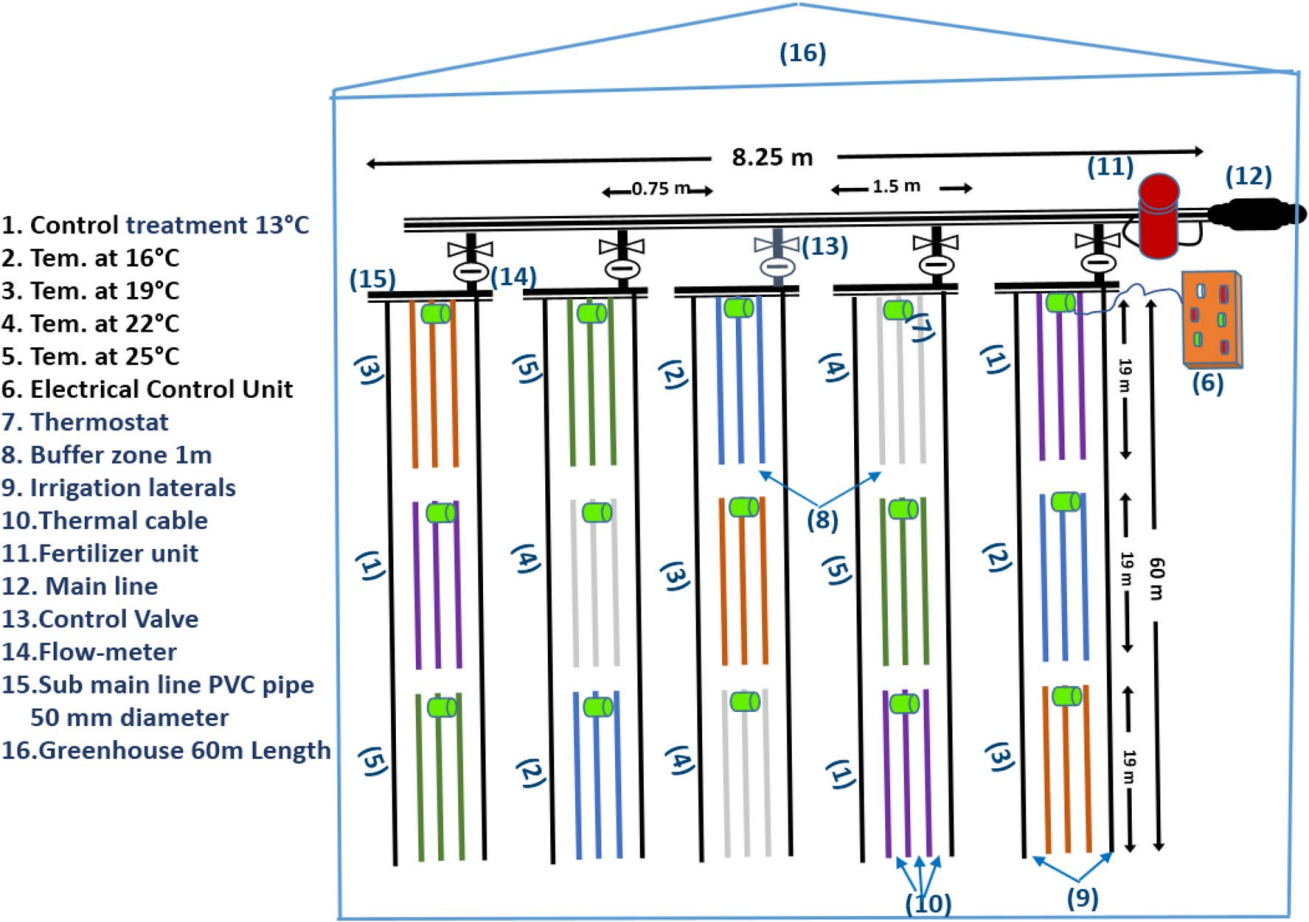
Experimental layout showing bed dimensions, irrigation laterals, the intelligent soil temperature control system, energy source, and the distribution of treatments and replicates.

Soil temperature was adjusted using a carbon fiber thermal cable with a metallic thermostat where, the apparatus of DR5003 made by National Company, Japan (Fig. 1-B). The thermal cable of 60 m was divided into three branches that fixed in parallel ten cm below the soil surface, which covered twenty meters long of the bed, and the distance was 20 cm between the branches, then buried and compacted well into the soil for each replicate (Fig. 1-C, 1&2). The thermostat was connected to a direct electrical source that was near to the greenhouse with an emergency circuit breaker. Cucumber plants were grown under five different soil temperature treatments (13 as a control, 16, 19, 22, and 25 °C) with three replicates per treatment in a completely randomized experimental design, with a 1 m buffer zone between replicates lined with rock wool as thermal insulation. The plot area was 20 m length and 1 m width (1/3 greenhouse bed) with two irrigation line spread above the bed and the distance between the plants was 50 cm, which contented 80 cucumber plants per each plot, as shown in the experimental layout Fig. 2.

The photosynthesis rate and stomatal conductance was determined using a portable photosynthesis meter (LI-6400, LI-COR, Lincoln, NE, USA). The measurements were performed on clear sky cover almost 11 AM, using the fifth top leaf with three plants for each replicate. The measurements were made on plant photosynthesis was obtained by multiplying the net photosynthesis rate by plant leaf area. Plant leaf area (cm^2^) was measured using a portable LI-3000 leaf area meter at 30, 45, 60, and 75 days post-transplanting, corresponding to four physiological growth stages: initial, development, mid-season, and late-season. Vapor pressure deficit (VPD) was estimated according to the meteorological data within the canopy area (temperature and relative humidity) for each treatment using the Extension Factsheet of^14^.

A sample of three plants was taken from each treatment replicate, after 60 days from transplanting, to determine the characteristics of plant height (cm) and number of leaves. Root density and distribution were determined by taken some of soil samples by hand auger from different distances and depths around the plant stem, thin washing to remove debris and soil particles, drying and collected the root system for weigh (g plant^−1^)^15^. Moreover, total chlorophyll content of the sixth mature leaf was determined using (Minolta chlorophyll meter Spad-501). Harvest initiated 60 days and was made twice per week. Fruit yield was measured as total yield kg plant^−1^. The yield during the first 15 days after harvest started was considered as early yield. The total soluble solids (TSS) was recorded using hand refractometer on sections taken from the central axis of the fruit^16^ while fruit firmness was measured using Magness and Ballouf pressure tester equipped with 3/16 inch^2^ plunger and adjusted in Newton (N). Available nitrogen, phosphorus and potassium content within 30cm soil profile was determined using Kjeldahl method described by FAO^17^ for nitrogen, using spectrophotometer according to^18^ for Phosphorus and using Flame photometer as described by Chapman and Pratt^19^ for Phosphorus. According to a comparison between two identical greenhouses, excluding the remaining heating systems (smart or conventional gas-fired systems at 19–22 °C for both systems), at the experimental site of the Egyptian Center for Protected Cultivation at the end of the experiment, the economic return was determined as follows:

Total annual costs: Fixed costs, greenhouse structural costs divided by 20 years, cooling and heating system costs divided by 10 years, irrigation system costs divided by 5 years, labor salaries during the germination and cultivation period, and variable costs, which include seedlings, fertilizers, other chemicals, and energy.

Net return: Calculated by subtracting the average estimated annual costs (C) from the average annual gross returns (B). To achieve the economic goal of maximizing net return, the system with the highest net return (B-C) is considered the most efficient based on economic analysis.

Benefit–cost ratio (B/C): Calculated by dividing the annual benefits by the annual costs. When aiming to maximize return on investment, the system with the highest benefit–cost ratio is considered the optimal choice according to economic analysis. It is common for one system to achieve the highest net return while another system achieves the highest benefit–cost ratio^20^ and the equations was follows:

$$Gross\;return\;(B,\$ ) = Crop\;yield\left( {ton\;per\;Standard\;greenhouse,520\;{\mathrm{m}}^{2} } \right) \times Price\left( {\$ ton^{ - 1} } \right)$$

$$Net\;return\;(B - C)(\$ ) = Gross\;return - Total\;annual\;costs$$

$$B/C\;Ratio(\% ) = Gross\;return/Total\;annual\;\,cost$$

To assess the effectiveness of the smart thermal system in saving energy, the thermal energy required to compensate for heat loss from the greenhouse body was calculated using the equation provided in^21^. The results were statistically analyzed using F-value test and the means were compared by the L.S.D at the level of 5% probability using MSTATC computer program. Levene test^22^ was run prior to the combined analysis to test the homogeneity of individual error terms. Duncan’s multiple range test was used at the 0.05 probability level to compare coefficient means.

## Results

### Physiological indicators

#### Net and plant photosynthesis rate

The treatments differed statistically (p < 0.05) in net and plant photosynthetic rate from the beginning of measurements, 30 days after the sowing date, until the end of 75 days of cucumber plants exposed to different soil temperatures C° (Table 3). Also in the same table, both net and plant photosynthesis showed an increasing trend with time, with a mean plant photosynthesis value of 3.2 µmol CO_2_ plant^−1^ s^−1^ at 30th days, 9.4 µmol CO_2_ plant^−1^ s^−1^ at 45th days, and 10.4 µmol CO_2_ plant^−1^ s^−1^ at 60th days, and then slightly decreasing at the end of plant growth at 75th days with an average value of 9.7 µmol CO_2_ plant^−1^ s^−1^ for the whole experimental season. The highest significant values recorded for the photosynthesis rate, whether for the plant or the net, were recorded at temperatures of 19 and 22 C°, with no significant differences between them during the different plant growth stages, except at 30th days (initial growth stage), where statistical significance appeared between them. At the same time, no significant differences were observed among the plants exposed to soil temperature at 13 or 16 C° at all of the plant growth stages, in both net and plant photosynthesis rate, except at 45th days in plant photosynthesis rate, where no statistical differences were observed between them. Moreover, the soil temperature treatment at 25 °C did not show statistical significance compared to the other soil temperature at 22 °C during the mid and late growing season in terms of photosynthesis rate only.

**Table 3:** Net and plant photosynthesis rates at 30, 45, 60, and 75 days after transplanting in cucumber under different soil temperature (C°).

Soil temperature C°	(µmol CO_2_ m^−2^ s^−1^) Net photosynthesis				(µmol CO_2_ plant^−1^ s^−1^) Plant photosynthesis			
	30 *days*	45 *days*	60 *days*	75 *days*	30 *days*	45 *days*	60 *days*	75 *days*
At 13	13.0 ± 0.44c	13.7 ± 0.61c	10.5 ± 0.36b	9.0 ± 0.61b	2.1 ± 0.10c	7.2 ± 0.12d	8.1 ± 0.40c	7.8 ± 0.21c
At 16	14.2 ± 0.41c	15.0 ± 0.73c	11.8 ± 0.40b	10.6 ± 0.28b	2.5 ± 0.08c	8.0 ± 0.10c	8.9 ± 0.57c	8.6 ± 0.34c
At 19	18.1 ± 0.32ab	21.4 ± 0.61a	14.6 ± 0.27a	12.1 ± 0.26a	3.6 ± 0.08b	10.6 ± 0.22a	11.6 ± 0.49a	10.4 ± 0.12ab
At 22	20.1 ± 0.15a	22.3 ± 0.52a	14.5 ± 0.48a	12.4 ± 0.31a	4.2 ± 0.12a	11.5 ± 0.18a	12.8 ± 0.26a	11.2 ± 0.19a
At 25	17.0 ± 0.58b	18.7 ± 0.58b	13.1 ± 0.61a	11.0 ± 0.55ab	3.3 ± 0.07b	9.5 ± 0.26b	10.8 ± 0.28b	10.2 ± 0.12b
LSD (0.05)	2.9	2.0	2.4	2.2	0.5	1.0	1.5	1.2

*Values are the mean of 3 replicates ± standard errors. Similar letters indicate nonsignificant at 0.05 levels using LSD least significant difference.

#### Stomatal conductance and vapor pressure deficit (VPD)

Significant changes in stomatal conductance of cucumber leaves or vapor pressure deficit conditions were observed at different soil temperatures  °C (Table 4). In this regard, the stomatal conductance of leaves gradually increases as the plant progresses until the development stage at 45th days, and then the trend begins to decline. The mean stomatal conductance values of 1.00, 1.31, 1.16 and 0.99 mol H_2_O m^-2^ s^−1^ at 30th, 45th, 60th, and 75th days, respectively. The values found that the conductance parameter at 22 C° recorded the highest significant value with 1.47 ± 0.09 at 30th days (initial stage) mol H_2_O m^−2^ s^−1^ compared to other treatments. In the development stage at 45th days, the same treatment and at 19 °C treatments recorded the highest significant values by values of 1.80 ± 0.06 and 1.80 ± 0.05 mol H_2_O m^−2^ s^−1^ respectively, with no significant difference between them. In mid-season at 60th days, the highest significant values were recorded with treatments of 19, 22, and 25 °C, with values of 1.53 ± 0.15, 1.57, and 1.57 ± 0.14 H_2_O m^−2^ s^−1^, with no significant differences between them. The same trend was found with the same treatments at the late season of 75th days, the values were 1.37 ± 0.07, 1.43 ± 0.03, and 1.23 ± 0.09 H_2_O m^−2^ s^−1^, respectively, with no significant differences between them. The conductance parameter values of treatments at 13 and 16 °C recorded no significant differences between them at all over the plant growth period.

**Table 4:** Stomatal conductance and vapor pressure deficit at 30, 45, 60, and 75 days after transplanting in cucumber under different soil temperatures (°C).

Soil Temperature C°	(mol H_2_O m^−2^ s^−1^ ) Stomatal conductance				kPa Vapor pressure deficit			
	30 days	45 days	60 days	75 days	30 days	45 days	60 days	75 days
At 13	0.87 ± 0.03c	1.03 ± 0.09b	0.90 ± 0.06b	0.70 ± 0.12b	0.93 ± 0.09a	1.01 ± 0.11a	1.85 ± 0.05a	2.32 ± 0.04a
At 16	0.93 ± 0.04c	1.13 ± 0.03b	1.00 ± 0.05b	0.83 ± 0.03b	0.91 ± 0.01a	0.90 ± 0.06a	1.77 ± 0.03a	2.30 ± 0.06a
At 19	1.27 ± 0.08ab	1.80 ± 0.05a	1.53 ± 0.15a	1.37 ± 0.07a	0.57 ± 0.02b	0.38 ± 0.01b	1.37 ± 0.12b	1.39 ± 0.01b
At 22	1.47 ± 0.09a	1.80 ± 0.06a	1.57 ± 0.14a	1.43 ± 0.03a	0.53 ± 0.03b	0.35 ± 0.02b	1.22 ± 0.04bc	1.38 ± 0.09b
At 25	1.23 ± 0.03b	1.75 ± 0.08a	1.47 ± 0.20ab	1.23 ± 0.09a	0.48 ± 0.02b	0.34 ± 0.02b	1.12 ± 0.07c	1.00 ± 0.10c
LSD (0.05)	0.23	0.35	0.49	0.4	0.2	0.28	0.24	0.37

*Values are the mean of 3 replicates ± standard errors. Similar letters indicate nonsignificant at 0.05 levels using LSD least significant difference.

The mean values of air vapor pressure deficit conditions were 0.60, 0.54, 1.26 and 1.46 kPa at 30th (initial), 45th (development), 60th (med), and 75th days (lite) season, respectively. There was a negative correlation between soil temperature and air vapor pressure; the data revealed that air vapor pressure deficit increased with decreased soil temperature °C all over the plant growth stages. The significantly highest values were recorded with treatments of at 13 and 16 °C, followed by at 19 and 22 °C, without significant differences between them in different growth stages, and finally the soil temperature at 25 °C, with no significant compared to the soil temperature at 22 °C in the med season, at 60th days only.

### Soil nutrients availability

Concerning the soil nutrients’ availability within the active root zone (Table 5), the trend shows that the N, P, and K availability were increased with increasing soil temperature. The highest significant values for the three macronutrient elements were recorded at a temperature of 22 °C and 25 °C, with values of 84.70 ± 1.56 and 89.18 ± 1.37 mg kg^−1^ soil, respectively, for nitrogen, 12.02 ± 0.12 and 13.23 ± 0.19 mg kg^−1^ soil for phosphorus, with no significant differences between them. However, potassium recorded the highest significant values only at 25 °C, with a value of 137.21 ± 0.90 mg kg^−1^. Soil temperatures at 13 and 16 °C recorded the lowest significant values for the three major macronutrients compared to the rest of the treatments, and there was no significant difference between those values. The soil temperature treatment at 19 °C showed moderately significant values for the availability of three macronutrient elements within the active root zone.

**Table 5:** Soil nutrients availability under different soil temperatures (°C).

Soil temperature C°	Soil macronutrients availability (mg kg^−1^ soil)		
	Nitrogen	Phosphorus	Potassium
At 13	56.32 ± 1.35c	05.94 ± 0.27c	092.03 ± 2.86d
At 16	58.21 ± 1.28c	07.17 ± 0.16c	094.49 ± 3.09d
At 19	66.02 ± 1.22b	09.22 ± 0.28b	110.73 ± 1.32c
At 22	76.23 ± 0.39a	12.02 ± 0.12a	124.41 ± 2.21b
At 25	80.26 ± 1.37a	13.23 ± 0.19a	137.21 ± 0.90a
LSD (0.05)	7.26	1.25	12.68

*Values are the mean of 3 replicates ± standard errors. Similar letters indicate nonsignificant at 0.05 levels using LSD least significant difference.

### Plant growth parameters

Plant growth parameters (Table 6), soil temperature at 22 °C produced the highest cucumber plants and the largest number of leaves per plant, and the maximum values of plant leaf area and the highest distribution of plant root systems, as well as the highest chlorophyll content in the leaf. This treatment yielded the highest significant values for these vital indicators during the plant’s progress. The recorded values were 208 ± 5.26 cm for pant height, 36.7 ± 0.38 leaves number, 8.77 ± 0.08 m^2^ leaf area, 7.73 ± 0.07 g root density, and 53.7 ± 0.35 Spad for chlorophyll content. The soil temperature treatments at 13 and 16 °C recorded the lowest significant values in all plant growth parameters, with no significant differences between those two treatments. At the same time, the soil treatments at 19 and 25 °C recorded moderate values with no significant differences between those two soil temperature treatments in all plant growth parameters, except root density showed no significant values between those soil temperature treatments of 19, 22, and 25 °C.

**Table 6:** Plant growth parameters in cucumber under different soil temperatures (°C).

Soil temperature C°	Plant growth characteristics				
	Height (cm)	No. of leaves	Total leaf area (m^2^)	Root density (g plant^−1^)	Chlorophyll Spad
At 13	183 ± 1.45c	26.0 ± 0.58c	7.72 ± 0.07c	4.80 ± 0.06b	45.4 ± 0.26c
At 16	188 ± 1.67c	28.3 ± 0.33c	7.88 ± 0.02c	5.23 ± 0.09b	45.5 ± 0.79c
At 19	217 ± 4.82b	33.7 ± 0.36b	8.53 ± 0.07b	7.33 ± 0.09a	51.0 ± 0.23b
At 22	245 ± 2.89a	36.7 ± 0.38a	8.77 ± 0.08a	7.73 ± 0.07a	53.7 ± 0.35a
At 25	208 ± 5.26b	31.7 ± 0.39b	8.41 ± 0.08b	7.30 ± 0.10a	50.1 ± 0.15b
LSD (0.05)	19	2.7	0.23	0.48	1.9

*Values are the mean of 3 replicates ± standard errors. Similar letters indicate nonsignificant at 0.05 levels using LSD least significant difference.

### Performance of cucumber yield quantity and quality

The performance of cucumber yield quantity and quality exposed to different soil temperatures was illustrated in Table 7. The yield quantity was total and early cucumber yield, while the quality was soluble solids solution and fruit firmness. The highest and largest cucumber significant yield was obtained with soil temperature treatment at 22 °C, with values of 3.71 ± 0.14, 0.88 ± 0.01, 13.37 ± 0.03, and 18.30 ± 0.10 for total yield and early yield kg per plant and TSS and fruit firmness, respectively. There were no significant differences in the values of quantity and quality of cucumber traits affected by soil temperature treatments at both 13 and 16 °C, while these two treatments recorded the lowest significant values compared to the other soil temperature treatments. In the same context, there were no significant differences in the values of productivity and quality traits of cucumbers exposed to soil temperature treatments at 19 and 25 °C, which recorded moderate values ​​in these traits.

**Table 7:** Performance of cucumber yield quantity and quality under different soil temperatures (°C).

Soil temperature C°	Yield			
	Quantity		Quality	
	Total yield kg plant^−1^	Early yield kg plant^−1^	TSS	Fruit firmness
At 13	2.14 ± 0.02c	0.41 ± 0.01c	11.07 ± 0.07c	12.40 ± 0.23c
At 16	2.33 ± 0.03c	0.42 ± 0.01c	11.43 ± 0.12c	13.23 ± 0.15c
At 19	3.27 ± 0.09b	0.77 ± 0.02ab	12.87 ± 0.13b	17.50 ± 0.29ab
At 22	3.71 ± 0.14a	0.88 ± 0.01a	13.37 ± 0.03a	18.30 ± 0.10a
At 25	3.25 ± 0.03b	0.76 ± 0.03b	12.50 ± 0.06b	16.97 ± 0.27b
LSD (0.05)	0.41	0.11	0.39	1.29

*Values are the mean of 3 replicates ± standard errors. Similar letters indicate nonsignificant at 0.05 levels using LSD least significant difference.

### Regression analysis

**Fig. 3:**
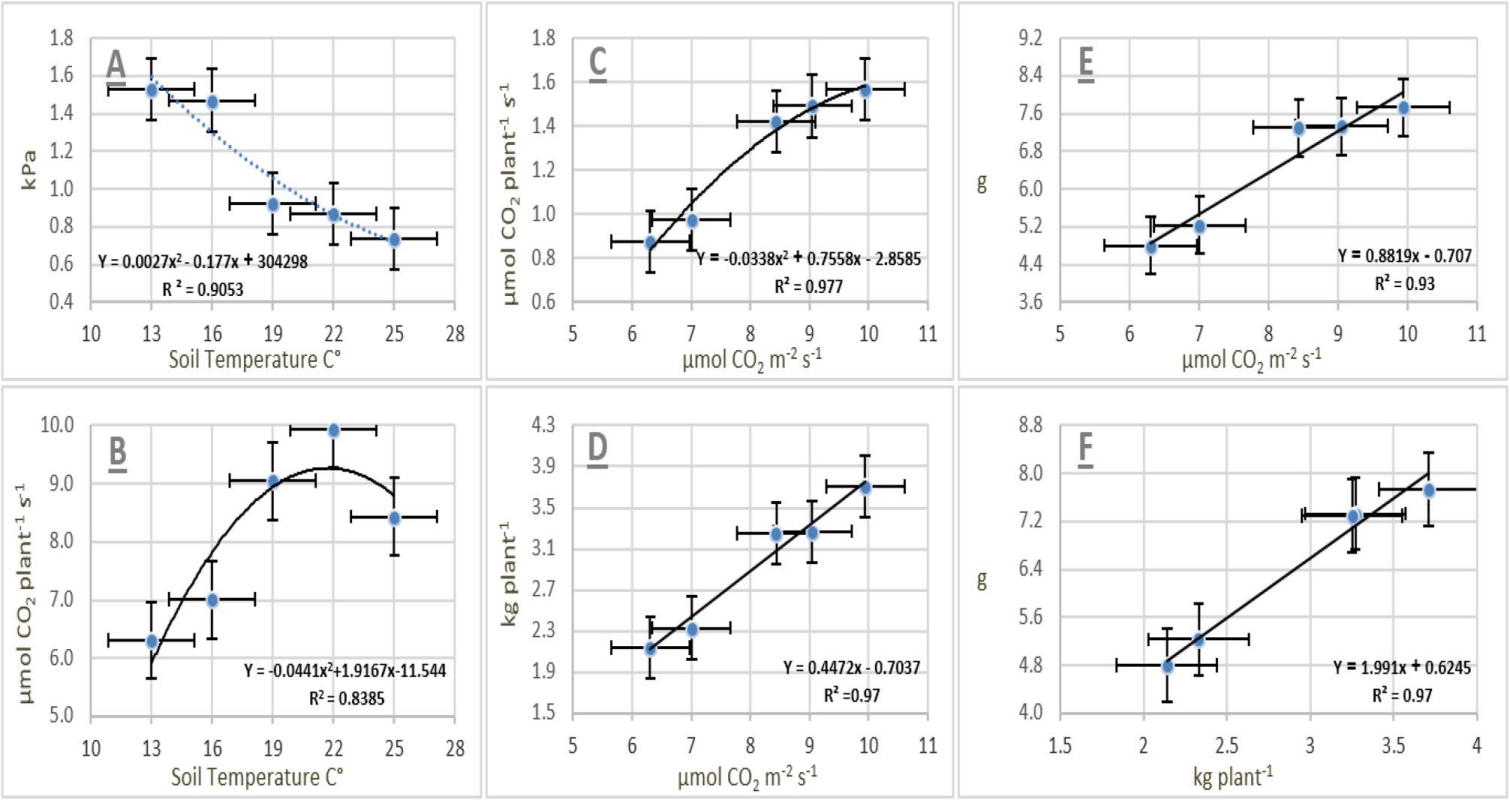
Linear and polynomial regression models describing the relationships between (**A**) soil temperature and vapor pressure deficit, (**B**) soil temperature and plant photosynthesis, (**C**) plant photosynthesis and vapor pressure deficit, (**D**) plant photosynthesis and total yield, (**E**) plant photosynthesis and root density, and (**F**) total yield and root density of cucumber grown under greenhouse conditions. All models exhibited high coefficients of determination.

Regression analysis was performed to evaluate the relationships between physiological indicators and crop parameters under different soil temperature treatments. The most appropriate functional forms describing these relationships are presented in Fig. 3. Regression equations were derived from the measured data and included both linear and polynomial models, selected based on the highest coefficient of determination (R^2^). Statistical analyses were conducted using Slide-write computer program. Figure 3A illustrates a polynomial relationship between soil temperature and average vapor pressure deficit (VPD), with a high coefficient of determination (R^2^ = 0.9053), indicating a decreasing trend in VPD with increasing soil temperature. In contrast, average plant photosynthesis exhibited an increasing polynomial response to soil temperature (Fig. 3B), with a strong model fit (R^2^ = 0.8385).

A strong polynomial relationship was also observed between average plant photosynthesis rate and stomatal conductance (Fig. 3C), with a high coefficient of determination (R^2^ = 0.9778), indicating that photosynthesis increased with enhanced stomatal conductance in cucumber leaves. Furthermore, a significant linear relationship was identified between average plant photosynthesis and total cucumber yield (Fig. 3D), with a high coefficient of determination (R^2^ = 0.972), demonstrating that increased photosynthetic activity was associated with higher yield. As plant photosynthesis integrates net photosynthetic rate and total leaf surface area, and its assimilates are allocated to specific organs such as the root system, a strong linear relationship was observed between plant photosynthesis and root density (Fig. 3E), with a high correlation value (R^2^ = 0.9322). Finally, total plant yield was strongly correlated with root density (Fig. 3F), showing a positive linear trend and a high coefficient of determination (R^2^ = 0.9772).

### Economic return of the smart thermal system

The following parameters were taken as an economical evaluation in case smart thermal system for heating standard greenhouse. The items of gross return LE and $, net return LE and $ and benefit–cost ratio (*B/C* ratio) were the major item in economic analysis.

Table 8 shows the gross return, net return, and benefit–cost ratio (*B/C* ratio). It’s clear that the gross return of smart thermal system was 203,750 (4075$) compared with the traditional heating system (greenhouse gas heater) which was 205,050 (4101$). The gross return of smart thermal system decreased by 0.64% compared with traditional heating system. While the net return of smart thermal system was 356,160 (7124$) compared with the traditional heating system which was 223,680 (4474$), an increase of 59.2%. Furthermore, the benefit–cost ratio of smart thermal system stood at 1.75%, surpassing the 1.10% of the traditional heating system. Economic analysis, which included criteria such as the benefit–cost ratio (B/C) and net return values (B-C), indicated that smart thermal system represented a viable economic option.

**Table 8:** Economic return of smart thermal system and traditional heating system.

Treatments	Gross return, LE ($)	Net return, LE ($)	*B/C* ratio, %
Traditional	205,050 (4101$)	223,680 (4474$)	1.10
Smart thermal unit	203,750 (4075$)	356,160 (7124$)	1.75

*B/C* = benefit–cost ratio.

### The effectiveness of the smart thermal system in saving energy

The combined monthly heat requirements for a cucumber crop grown in a standard 520 m^2^ greenhouse for both conventional and smart heating systems are presented in Table 9. The data shows the energy savings in the smart heating system, which can reach up to 30% of the cucumber crop’s requirements, depending on the growing season, region, and wind direction. Heat requirements vary throughout the growing season, peaking during November and January, when the plant begins to grow and develop.

**Table 9:** Heat requirements for a 520 m^2^ plastic cucumber greenhouse (kcal /season) of 2022 at the Giza–Dokki experimental site.

Heating system	November	December	January	February	Total
Traditional	13,230,450	9,671,850	10,128,150	8,260,700	41,291,150
Smart thermal unit	9,261,315	6,770,295	7,089,705	5,782,490	28,903,805

## Discussion

It is very important to achieve the optimum soil temperature to understand its effect on physiological indicators, and know the source-sink relationship to clarify the response mechanisms of the cucumber crop growing under protected cultivation conditions^23^. The sink crop organ can limited by the photosynthesis supplying from crop leaves source or by the ability of sinks to benefit from photosynthesis^6,24^. The obtained results confirmed that deviation from the optimum soil temperature, whether increased or decreased, exposes the plant to abiotic heat stress that negatively affects either net or plant photosynthesis rate, the stomatal conductance then affects growth and yield of cucumber. The soil temperature at 22 C° led to increases in both the net and plant photosynthesis rate, stomatal conductance, during the different critical growth stages. Also, the same soil temperature within the active rood zone enhancing the soil nutrients availability N, P and K which was reflected in plant growth parameters (plant height, number of leaves, total leaf area, root density and chlorophyll content). Regarding the water vapor pressure values, it was increased with decreased soil temperature within the active root zone. The physiological and morphological growth parameters were achieved with the ideal soil temperature, which enhanced the high productivity (total and early yield) and suitable quality (TSS and fruit firmness) of the cucumber crop.

During photosynthesis, the CO_2_ fixed reaction is an enzyme reaction strongly controlled by temperature^25^, thus the reduction rate of photosynthetic at the late season could be attributed to reducing the solubility of CO_2_, photorespiration, senescence, and increased diffusion resistance. Also, temperature stress might be inhibition of photosynthesis and damage the mechanism of the CO_2_ fixed reaction^26–28^. This implies that heat disrupts cellular balance, causing reactive oxygen species overproduction, which signals distress or causes harm, related to heat stress directly to oxidative stress^23,29^. Stomatal conductance is controlled by two ways, instantaneous response (aperture of stomatal pores) and the long term response (size and number of stomatal within leaf) as^4^ pointed out. So, cucumber plants exposed to optimal soil temperature at 22 °C (long term response), show larger stomata and greater stomata density^30^ Furthermore, stomatal response to change in air current of the leaf boundary layer can be conjectured to relate to changes in the intercellular CO_2_ concentration and the transpiration water loss of the leaf^31^. According to standard ISO^32^, water vapor pressure values between 0.4 and 1.4 kPa are considered the optimal humidity for plant growth as well as yield quantity and quality, these values were recorded with a soil temperature of 19 °C and above during the critical growth stages of the season. Continuous vapor pressure deficit can induce calcium deficiency within the active root zone and that teat to reducing the plant leaf area, root density, as well as chlorophyll content and other growth parameters in various greenhouse vegetables^33^ and^34^. Several studies have shown that as soil temperatures rise, available nutrients become more readily available in greenhouse crops^10,35,36^. This may be because soil water retains heat as latent heat. Temperature also affects the rate of chemical reactions in the soil, affecting nutrient availability. This may be because higher temperatures promote mineralization, leading to increased nutrient availability and enhanced plant uptake. Temperature can also affect the solubility of nutrients, affecting their availability to plants^37^ This may explain the response of nutrient availability to rising soil temperature under the study. Under environmental stressors, the plant cells produce excessively reactive oxygen species^38^, that can destroy the cell membrane and other organelles such as chlorophylls, which leads to losing vital cell functions, thus the content of the antioxidants enzymes was enhancing. Thus, heat stress, both high and low had affected all plant physiological indicators and this was reflected in growth and productivity. Optimal temperature stimulated plant growth parameters and increased the early and total yield of various greenhouse vegetables^10,39^. The main benefits of optimal soil temperature include earlier crop harvest, improved total soluble solids (TSS), and firmness of cucumber fruits. This may be due to the system provides a more stable root zone temperature during critical nighttime periods, which influences the microclimate surrounding the plants^23,39^. Furthermore, physiologically, cucumber yield appears to depend on the balance between photosynthesis and respiration; therefore, soil and air temperature, and soil moisture play a significant role in cucumber yield and quality^33^. This is clearly evident from the relationships between the various physiological indicators, which are characterized by a high correlation coefficient, both among themselves and with soil temperature. The economic analysis shows that the cost of traditional heating systems was higher due to the need for an internal air heating compressor and the additional cost of gas energy required to operate it, while this was absent in the smart heating system. The additional revenues added through increased crop productivity in the smart heating system allowed for greater profitability as shown by the increased net return and higher benefit–cost ratio as^20,40^. It is well known to greenhouse growers that, for cucumbers in particular, if nighttime temperatures drop two or three degrees below the optimum for plant growth for several hours, this leads to an irreversible reduction in growth and productivity^21^. The smart heating system used has saved approximately 30% energy compared to the traditional system. This may be due to the lack of need for continuous monitoring of operation and shutdown of the system, which usually occurs late at night or early in the morning. The traditional system is always left on for several hours or starts up early due to human intervention. On the other hand, monitoring the greenhouse temperature and smart operation as soon as the temperature drops below the optimum level and does not allow it to reach zero °C, thus preventing harmful fluctuations in temperature.

## Conclusion

Based on the physiological indicators assessment of photosynthesis, stomatal conductivity, and vapor pressure deficit, it was found that temperatures lower than 19 °C or higher than 22 °C during different growth stages of cucumber plants had a negative effect on growth parameters and yield indicators. This was confirmed by the regression analysis relationships obtained when comparing the physiological factors with the different soil temperatures under study, as well as between them and root distribution, and yield. Furthermore, it was evident that higher soil temperatures increase nutrient availability within the effective root zone. Therefore, a soil temperature of 22 °C can be recommended as an acceptable soil temperature for cucumber growers at the beginning or middle of the season under similar conditions. However, at the end of the season, the plant is not affected, so a soil temperature of 19 °C can be used to save energy. Furthermore, from an economic and energy-saving perspective, we recommend the use of smart thermal sustainability systems for heating to improve the productivity and quality of greenhouse cucumbers on cold days, at night, or in the early morning, further achieving Egypt’s 2030 Sustainability Plan and beyond.

## Data Availability

All data supporting the findings of this study are available within the paper and the attached file.
